# Exploring Creative Entrepreneurs’ IEO: Extraversion, Neuroticism and Creativity

**DOI:** 10.3389/fpsyg.2020.02170

**Published:** 2020-08-28

**Authors:** Yang Gao, Dixuan Zhang, Hongjia Ma, Xiaomin Du

**Affiliations:** ^1^School of Economics and Management, Dalian University of Technology, Dalian, China; ^2^School of Management, Jilin University, Changchun, China; ^3^School of Economics and Management, Yingkou Institute of Technology, Yingkou, China

**Keywords:** IEO, creativity, extraversion, neuroticism, entrepreneurship

## Abstract

In creative industries, entrepreneurs’ creativity is the source of entrepreneurial activities. Meanwhile, the key to the success of entrepreneurship lies in the strategic tendency of leaders, referred to the individual entrepreneurial orientation (IEO). This paper is aimed to explain the process from creativity to IEO and reveal the psychological process of entrepreneurs’ creativity. We proposed an integrated model of “individuality–creativity–IEO” by drawing on the theories of big five personality and entrepreneurship. Using a sample of 202 creative entrepreneurs from China, the research found that entrepreneurs’ creativity has a positive impact on IEO, and the individuality of neuroticism and extraversion exhibited a negative U-shaped relationship with creativity. Finally, implications for both theory and practice were presented.

## Introduction

Creative industries, one of the success stories of the 21st century (Bilton, 2007), are the driving force of employment creation, regional innovation and social integration (Henry and de Bruin, 2011). Creative industries are different from traditional industries in that most creative enterprises are in the initial stage of development and are small in scale. Moreover, these enterprises are mainly characterized by the labor input of creative entrepreneurs and utilize the unique artistry and originality of their products or services as competitive advantages. The uniqueness of these industries mainly comes from the creative characteristics and innovative ability of the entrepreneur as a designer, artist, etc. (Ellmeier, 2003; Chaston and Sadler-Smith, 2012). Therefore, creative entrepreneurs’ innovative ability and strategic preference are key to success. However, although some studies have paid attention to the importance of creative entrepreneurs, there is still a lack of in-depth discussion on the relationship between creativity and entrepreneurial orientation.

For creative industries, according to Hambrick and Mason’s (1984) upper echelon theory, the strategic tendency and choices of leaders determine the entrepreneurial behavior of firms, thereby determining the entrepreneurial performance. Therefore, the key to the success of creative industry entrepreneurship lies in the strategic tendency and choices of the leaders, which is commonly described as the individual entrepreneurial orientation (IEO). However, current research on entrepreneurial orientation is based on the firm level but not the individual level (Lumpkin and Dess, 1996; De Clercq et al., 2010; Bolton and Lane, 2012). These studies primarily analyzed the impact of entrepreneurial orientation on entrepreneurial success and entrepreneurial performance, but there is still a certain gap in the research on how individual entrepreneurial orientation is generated (De Clercq et al., 2010; Gupta et al., 2016). In particular, in creative industries, the core of entrepreneurial activities is creativity (Ward, 2004). Creativity is the source power for the sustainable development of creative industries. Without creativity, entrepreneurial activities will become worthless. According to Baum and Locke (2004), creativity is similar to a raw material, it can be stressed through the process of entrepreneurship and strategic decision making. Therefore, it is important to explore how creativity affects individual entrepreneurial orientation. However, there are few empirical studies on the antecedents of individual entrepreneurial orientation and how they are affected by creativity. Therefore, the first research question this paper seeks to answer is that how individual entrepreneurial orientation in creative industries is affected by creativity.

Creativity refers to the ability to generate new or useful ideas (Ward, 2004). The motivation for creative individuals to choose entrepreneurship lies in the fact that they can show their creativity and realize their novel and unique ideas while starting a new business (Ellmeier, 2003; Chaston and Sadler-Smith, 2012). To understand why the individual creativity of entrepreneurs can affect their own entrepreneurial orientation to different degrees, we will continue to explore how the individual creativity of entrepreneurs is stimulated. Based on relevant theories of creativity (Hammond et al., 2011), scholars have noted that personality traits are quite predictive of creativity and are often lasting. These characteristics distinguish those individuals who are more likely to produce creative output from those who are not, and affect creativity by reducing the behavior threshold (Hao et al., 2016). Especially in creative industries, the creative personality of creative entrepreneurs is an important source of value creation, so we will explore how it works as a independent variable of creativity. Previous studies investigating the influence of entrepreneurs’ personality characteristics on creativity focused on big five personality and obtained relatively consistent conclusions (Furnham and Bachtiar, 2008; Feist, 2011). However, controversy remains regarding whether these results can be applied to creative industries. According to Chaston and Sadler-Smith (2012), the personality of entrepreneurs in creative industries is different from that of traditional entrepreneurs: creative entrepreneurs have some unique personality traits, especially in terms of neuroticism and extraversion (Chen and Chen, 2012; Chiang et al., 2015; Scott and Nicos, 2015). In the Cambridge Handbook of Creativity, Feist (2011) proposed that, in addition to the previously mentioned traits, including cognitive traits, social traits and motivational-affective traits, clinical personality traits like neuroticism is another factor that affect creativity, which is consistent with Eysenck’s (1990) discovery. Eysenck (1990) asserts that neuroticism is the personality dimension most closely related to creative thinking and behavior. For extraversion, scholars have come to different conclusions on the influence of extraversion on creativity, wherein the results indicated positive effects, negative effects or even no effect (Batey et al., 2010; Kandler et al., 2016). To solve this argument, we use the theory of big five personality for reference to propose the second research question: how do neuroticism and extraversion characteristics of creative representatives affect individual creativity differently.

**FIGURE 1:**
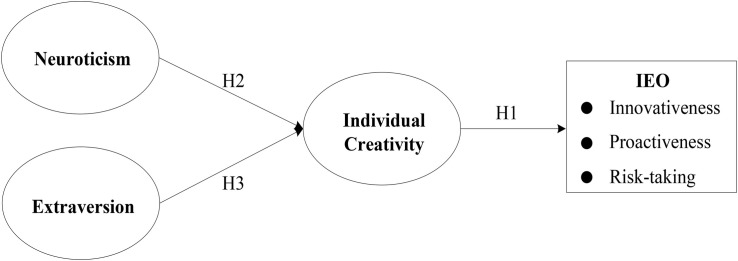
Conceptual framework.

The IEO of creative entrepreneurs determines the success of entrepreneurial behavior, and success is often reflected in the innovative creativity of creative entrepreneurs. Therefore, for creative industries, it is necessary to explore the influence of creativity on IEO and further research what factors can lead to creativity. Based on the theories of entrepreneurship management and psychology, we propose an integrated model from creativity to entrepreneurship at the individual level to predict the entrepreneurial behavior of entrepreneurs. First, we seek to fill the gaps in existing research on individual entrepreneurial orientation by responding to the call of Bolton and Lane (2012) for further discussion on the dimensions and antecedents of IEO. Second, drawing upon the theories of big five personality and creativity, our research explores the influence of entrepreneurs’ extraversion and neuroticism on creativity by combining interdisciplinary research. Finally, we propose inverted U-shaped relationships between neuroticism and creativity and between extraversion and creativity, thereby filling the gaps in the existing research on non-linear relationships in the psychology domain. Figure 1 shows the conceptual framework of this study.

## Literature Review and Hypotheses

### Individual Entrepreneurial Orientation

Entrepreneurial orientation is a widely used concept in entrepreneurship theory. Scholars have verified its influence on entrepreneurial performance, profitability and product innovation (Wiklund and Shepherd, 2005; Semrau et al., 2016). However, most previous studies on entrepreneurial orientation were performed at the firm level. Recently, scholars found that—according to the upper echelons theory—the EO of individual members can decisively predict the entrepreneurial results and help shape important firm outcomes. These studies were led by researchers, including Ferreira et al. (2015) and Keil et al. (2017), to empirically investigate the EO of key decision makers in firms (i.e., CEOs), which corresponds to the level of the individual. In addition, as stated by Williams (2009), the essence of entrepreneurship lies in those entrepreneurs who habitually create and innovate. They create value with perceived opportunities and promote organizational development and economic growth with risk-taking spirit and innovative ability. Especially in creative industries, the core of entrepreneurship is the labor input of creative entrepreneurs. The way in which creative entrepreneurs make innovative strategies sets them apart from traditional entrepreneurs: their investment in the environment is to create symbolic recognition, not material interests. Therefore, entrepreneurial orientation at the individual level may have implications not only for explaining what kind of entrepreneurial strategic choices creative entrepreneurs take to achieve entrepreneurial success but also for explaining how their entrepreneurial strategic preference is different from that in other industries (Kollmann et al., 2017). A distinguishing characteristic of creative entrepreneurs is that their free lifestyle tends toward new risk creation, and they devote themselves to art for the sake of art (Eikhof and Haunschild, 2006; Wu et al., 2018; Yuan and Wu, 2020). Creative artists and creative entrepreneurs decide to start their own businesses because entrepreneurship can embody their interests, skills and talents in their work (Paige and Littrell, 2002). Additionally, this is the difference between creative entrepreneurs and entrepreneurs in other industries.

Based on the EO’s existing theories, we make the definition of IEO as key entrepreneurs’ strategic orientation, that is, IEO reflects the strategic preference of entrepreneurs to carry out higher level innovation activities, take the initiative to risks and actively defeat competitors (Bolton and Lane, 2012; Goktan and Gupta, 2015). Recently, scholars found that–according to the upper echelons theory–the IEO of entrepreneurs can decisively predict the entrepreneurial behavior and results, also, can help shape important firm outcomes. The study of entrepreneurial orientation at the individual level provides a new research level and perspective for entrepreneurial researchers. The existing research on IEO holds the view that the individual entrepreneurial orientation is a multidimensional construct composed of elements similar to the entrepreneurial orientation at the firm level. Bolton and Lane (2012) recently proposed, developed and verified the dimensions of IEO. Through empirical research, they have shown that IEO is composed of innovativeness, proactiveness and risk-taking, which has been widely used by IEO researchers. For example, the behavioral characteristics model developed by Frese and Gielnik (2014) asserts that compared with other behavioral characteristics, IEO has a higher correlation with corporate performance. Additionally, Chien (2014) found that the IEO of Taiwan’s franchisors was positively correlated with business performance. A relationship between IEO and business success was also reported by Bolton and Lane (2012). The above studies have indeed given some new insights on IEO at an individual level of EO However, most studies focused on the IEO-performance relationship. Since IEO exists at the individual level, its relationship with personal environment, personality, entrepreneurial attitudes and other factors is also worth researching (Zhao et al., 2011; Ferreira et al., 2015).

### Individual Creativity and IEO

Creativity refers to the ability to generate novel or useful ideas (Ward, 2004). Previous research has recognized the importance of creativity to entrepreneurship. For example, Biraglia and Kadile (2017) claim that presenting novel and useful ideas is valuable for discovering opportunities and starting new businesses. Khedhaouria et al. (2015) assert that entrepreneurship is a process driven by leaders and triggered by personal creativity and self-efficacy. Sternberg (2004) suggests that the ability to think outside of one’s own mind may affect the strategic choice to form a new enterprise. Creativity is more important for creative industries that create value based on novelty and uniqueness. Novel and useful ideas are the lifeblood of entrepreneurship (Ward, 2004). The creative industry relies on the artistry and originality of their products or services to attract the attention of potential customers and achieve initial market success (Ellmeier, 2003; Henry and de Bruin, 2011), and this uniqueness is mainly derived from the creativity of creative entrepreneurs (Shane and Nicolaou, 2015). Starting a new business gives creative entrepreneurs more autonomy to become inspired and unleash their imagination to help realize what they want to achieve in the creative industry. According to Bolton and Lane (2012), IEO has been suggested to have three subdimensions: innovativeness, proactiveness and risk-taking. Innovativeness refers to the willingness to support creativity and experimentation in introducing new products or services and novel, technological leadership and R&D in developing new processes. To explore and develop new ideas, creative entrepreneurs need to lead companies to adopt innovative and proactive strategies (Puhakka, 2013). Without creative ideas, the entrepreneurial process lacks raw materials (Yuan and Woodman, 2010; Scott and Nicos, 2015). Baer (2012) argues that creativity provides the basis for innovation. Identifying an individual’s creative potential is critical to entrepreneurial success. Proactive creative entrepreneurs are continually seeking insights from current trends and future opportunities, so they may inherently develop persistent concentration on the task at hand, thereby giving themselves a better chance to achieve creative ideas (Chen et al., 2015). Individual creativity resulting in novel ideas would need proactiveness to extend the idea into implementation or to be adopted as an innovation (Feldman and Bolino, 2000). According to Covin and Miller (2014), risk-taking propensity is reflected in an entrepreneur’s strategic actions in the face of uncertainty. Thus, risk-taking refers to the willingness to act boldly to seize opportunities even if there is no guarantee of success (Chen et al., 2015). For creative industries, entrepreneurs invest under extremely uncertain circumstances to create symbolic recognition, to dedicate themselves to art rather than material gain (Eikhof and Haunschild, 2006). Risk-taking reflects the extension of idea implementation or adoption and requires initiatives such as risk-taking to expand idea diffusion, and one distinguishing characteristic of creative entrepreneurs is their lifestyle-driven tendency toward new venture creation (Eikhof and Haunschild, 2006). We propose the following hypothesis:

Hypothesis 1a: IC is positively related to innovativeness.

Hypothesis 1b: IC is positively related to proactiveness.

Hypothesis 1c: IC is positively related to risk-taking.

### Personality and Individual Creativity

For a better understanding of how creativity affects the IEO of creative entrepreneurs, it is important to explore what antecedents may stimulate creativity. Based on the theory that creativity is primarily determined by stable traits, these traits are quite predictive of behavior and are always persistent. Therefore, these characteristics distinguish those people who are more likely to produce creative output from those who are not and influence creativity by reducing behavior thresholds. Startup companies in creative industries heavily rely on the artistry, uniqueness, and originality of their products or services to attract the attention of potential audiences and achieve initial market success (Ellmeier, 2003; Henry and de Bruin, 2011; Wu et al., 2020). Thus, it is important to explore the influence of their personal characteristics on creativity. Previous studies on these influences focused on the big five personality. As an integrated conceptual framework of a few comprehensive categories including all traits, big five personality theory covers explicit facets of trait research, so we take it as a theoretical perspective of trait research. Previous studies on the impact of big five personality traits on creativity reached relatively consistent conclusions: higher openness and extraversion and lower agreeableness, conscientiousness, and neuroticism have positive effects on creativity. However, controversy remains regarding whether these findings can be applied to creative entrepreneurs. Chaston and Sadler-Smith (2012) asserted that the personality of entrepreneurs in the creative industry is different from that of traditional entrepreneurs. Creative entrepreneurs have some unique personality characteristics, especially in two aspects of creative entrepreneurs: neuroticism and extraversion (Batey et al., 2010; Chen and Chen, 2012; Chiang et al., 2015; Scott and Nicos, 2015). For neuroticism, Eysenck claims that neuroticism is the personality dimension most closely related to creative thinking and behavior. However, at present, scholars have different opinions on the influence of neuroticism on creativity. For instance, some studies found a negative association between neuroticism and creativity (Guo et al., 2017). In contrast, others have found that self-rated creativity is positively related to neuroticism (Batey et al., 2010). However, other researchers have found no significant relationship between neuroticism and creativity (King et al., 1996). Thus, the association between neuroticism and creativity seems complex. For extraversion characteristics, scholars have found that the influence of the extraversion of creative industry entrepreneurs on creativity is different from the previous conclusions. For instance, Zhang et al. (2017) argued that introversion is needed for creativity since it requires an introspective process that requires time alone. However, other researchers have suggested that extroverted entrepreneurs are energetic, enthusiastic and divergent thinkers that can improve their creativity. Therefore, it seems that the findings regarding the relationships between personality and creativity are inconclusive (Zhou and Hoever, 2014). The specific impact of neuroticism and extraversion on the creativity of creative entrepreneurs will be discussed in detail in the following sections.

#### Non-linear Relationship Between Extraversion and Individual Creativity

In some studies, extraversion was found to be associated with creativity since it is associated with stimulus seeking and creative thinking (Srivastava and Ketter, 2010; Kandler et al., 2016). However, other studies found that introversion is needed for creativity, since creativity requires an introspective process that requires time alone (Feist and Barron, 2003; Feist, 2011). In that sense, several scholars have provided theoretical arguments supporting both a positive and a negative relationship between extraversion and individual creativity. Extroverted entrepreneurs are open-minded, energetic and intelligent. They often reflect on their ideas to assess and define problems and opportunities and then develop solutions through their creativity and divergent thinking. Raja et al. (2004) assert that extraversion reflects the positive tendency of individuals to be energetic, enthusiastic and ambitious. Creativity may come from a person’s positive behavior, actively participating in tasks or trying different ideas. For this reason, those who passively wait for others to inspire and stimulate them are less likely to be creative. Extroverts tend to seek new ways of working and face problems rather than avoiding them, which may improve their creativity. According to Sung and Choi (2009), extraversion may be the most important predictor of creativity performance among the five factors. Indeed, highly extroverted people are full of energy and enthusiasm, encouraging behaviors such as seeking stimulation and solving problems, which should enhance creative thinking and performance (Costa and McCrae, 1992; Furnham and Nederstrom, 2010). In addition, research shows that advertising professionals, designers and artists usually score high on extraversion (Srivastava and Ketter, 2010). Extroverts may use divergent thinking test scenarios as a way to find excitement, and they may be more willing to work with others to improve creativity (Furnham and Bachtiar, 2008; Srivastava and Ketter, 2010; Kandler et al., 2016). However, some people hold the opposite view. Feist (2011) asserts that socialization is an aspect of extraversion, which has a complex relationship with artistic and scientific creativity, especially for creative high achievers who need much time to think and elaborate ideas alone. In addition, creative people, especially in the fields of art and science, often have a stronger desire than normal people to liberate themselves from social interactions and be overwhelmed by the stimulation of novel social situations. In fact, an excessive principle of creative thinking and behavior is its relatively non-social or even antisocial orientation. To be creative, one must be able to be alone and away from others. The creative process usually requires solitude (Feist and Barron, 2003). Runco (2010) suggests that creative people pay more attention to the inner world of thought. Therefore, introversion rather than extraversion is more likely to predict creativity.

To reconcile these opposing views, our study adopts the “too much of a good thing” (TMGT) meta-theoretical framework (Pierce and Aguinis, 2013) from the psychology domain. The TMGT effect describes situations in which “ordinarily beneficial antecedents reach inflection points, after which their relations with the desired outcomes cease to be linear and positive” (Pierce and Aguinis, 2013). Similarly, we propose that the relationship between extraversion and individual creativity is curvilinear, with individual creativity decreasing after exceeding a certain threshold of extraversion. When creative entrepreneurs are at a lower level of extraversion, people will actively become curious about the outside world and generate novel ideas as their extraversion increases. This active contact with the outside world inspires the use of divergent thinking to solve problems, thereby improving creativity. However, given that creative entrepreneurs spend much time and energy on external things when extraversion is at a high level, they lack sufficient time for thinking and elaborating ideas, which affects their attention to the inner world and subsequently lowers their generation of creativity. Therefore, we propose hypothesis 2, which is described hereafter.

Hypothesis 2: The relationship between extraversion and individual creativity is curvilinear (inverted U-shaped), wherein excessive introversion or extraversion have a negative correlation with individual creativity and a moderate level of extraversion has a positive correlation with individual creativity.

#### Non-linear Relationship Between Neuroticism and Individual Creativity

The relationship between neuroticism and individual creativity is puzzling. Although some researchers have found that self-rated creativity is positively related to neuroticism (Batey et al., 2010), other researchers (Chamorro-Premuzic and Reichenbacher, 2008) found negative effects, especially under some stimulating conditions. In especially under some stimulating conditions. In addition, some researchers found the relationship no significant (King et al., 1996).

Eysenck (1990) asserts that neuroticism is the genetic advantage of creativity, mainly through its relationship with low cognition and behavior inhibition, leading to higher conceptual fluency and originality and increased independence and normative challenging behavior. Creative entrepreneurs are more anxious, emotional and sensitive, whereas scientists tend to be more emotionally stable. Neuroticism will lead creative people to choose an art field to express themselves, or neuroticism is truly an art-promoting factor. Hence, this factor leads to higher achievements through higher sensitivity to emotional stimulation, promoting the exchange of emotional ideas in art works (Feist, 1998; Batey and Furnham, 2006). In addition, Srivastava and Ketter (2010), through meta-analysis, found that neurotic creative business professionals are more creative than non-neurotic business professionals. Some scholars claim that emotionally stable individuals with low neuroticism are relatively relaxed and have a positive view of the tasks of themselves and others (Guo et al., 2017). Creativity requires the ability to effectively integrate information and seek a new way of thinking, which can be enhanced by a calm attitude and self-confidence. Therefore, entrepreneurs with high emotional stability are more willing to participate in the difficult process of creative problem solving (Batey et al., 2010).

Neuroticism has both positive and negative effects on individual creativity, indicating that the relationship between neuroticism and individual creativity, according to the “ubiquitous U” principle from the psychology domain (Landon and Suedfeld, 1972), is curvilinear. Hence, when entrepreneurs’ neuroticism is at a low level, neuroticism and creativity are positively related. With the increase in neuroticism, creative entrepreneurs will show higher originality and artistry. However, to some extent, these influences will become less positive (diminishing marginal utility) and negative (non-monotonous). Because emotions are too sensitive and unstable, excessive anxiety and emotional situations may occur, affecting their thinking processes and ability to integrate information, ultimately reducing creativity. Therefore, we propose hypothesis 3, which is described hereafter.

Hypothesis 3: The relationship between neuroticism and IC is curvilinear (inverted U-shaped), wherein high emotional stability and excessive neuroticism have a negative correlation with IC and a moderate level of neuroticism has a positive correlation with IC.

## Materials and Methods

### Research Design and Data Collection

Our research adopts the definition of creative industries made by the United Kingdom’s Department of Culture, Media, and Sport (DCMS, 2001), and entrepreneurs whose firms engage in the following creative businesses are chosen as the target sample: art, craft, design, fashion, filming, advertising, architecture, publishing, media and cultural heritage. Participants in this study are entrepreneurs who are founders and are currently in charge of creative businesses in China. In the sample selection, we identified potential samples from the National Small and Medium-sized Enterprise Service Platform managed by the Ministry of Industry and Information Technology of the People’s Republic of China. There is a large number of directories and a substantial amount of basic information on the creative enterprises in this platform. To ensure sample representativeness, we select enterprises located in areas with different levels of economic development (including the eastern, central and western economic zones of China), which can avoid the influence of regional economic development on the study. And according to Comrey and Lee (2013) stated, a sample size of 200 is reasonable, 300 is good. Therefore, the potential sample in this study was set as around 300.

The data were collected in three phases. In the first phase, we collected 100 cases from each of the three regions: the eastern, central and western economic zones of China (a total of 300 enterprises). Their contact information was obtained through Tianyancha, a professional enterprise credit query platform in China. The second step was to contact the creative firms and CEOs, general managers or owners who were the leading founder of each firm by phone, email, and other means of communication. At the beginning of the survey process, we specified the purpose of the survey, stated that our research was sponsored by the National Social Science Foundation, and guaranteed that the survey data would be kept confidential and be used only for academic research. Finally, we added their email addresses, WeChat (a chat app in China) and other communication channels so that official questionnaires could be distributed through the network. The next step was to send formal questionnaires online. We sent the questionnaire link through the questionnaire collection tool to the entrepreneurs by email, WeChat and other communication channels. The data collection process took 7 months, from June 2019 to January 2020, during which 264 questionnaires were completed. However, to ensure the high quality and validity of the questionnaire, 62 invalid questionnaires were deleted, including those with interruptions, short filling times (less than 5 min), vacancies or invalid information. Finally, we used 202 high-quality valid questionnaires. Considering the different sources of samples, it is necessary to verify whether they can be used together. In this study, we use one-way ANOVA test to verify whether there are differences in the main research variables among the eastern, central and western economic zones of China. The results showed that there was no significant difference in the items of the main variables involved in the questionnaire from different sources (*P* > 0.05), indicating that the samples from the three sources were basically from the same matrix and could be used together. Table 1 shows the sample characteristics. Among the respondents, 69 (34.2%) were less than 30 years old, 65 (32.2%) were between 31 and 35 years old, 25 (12.4%) were between 36 and 40, 13 (6.4%) were between 41 and 45, and 30 (14.9%) were more than 46 years old. Moreover, 109 (53.96%) respondents were male, whereas 93 (46.04%) were female. By specialization, 58 respondents were in software (28.7%), 35 in advertising (17.3%), 21 in filming and TV (10.4%), 19 in design (9.4%), 18 in music and picture (8.9%), and 15 in art (7.4%). In terms of educational background, 22 (10.9%) respondents had a high school diploma, 84 (41.6%) had a bachelor’s degree, 53 (26.2%) had a master’s degree, and 15 (7.4%) had a doctorate.

**TABLE 1:** Characteristics of the research samples (*N* = 202).

Control variables	Item	Frequence	Percentage	Control variables	Item	Frequence	Percentage
Gender	Male	109	54.0	Industry	Advertising	35	17.3
	Female	93	46.0		Design	19	9.4
Age	≤30	69	34.2		Software	58	28.7
	32–35	65	32.2		Filming and TV	21	10.4
	36–40	25	12.4		Music and Picture	18	8.9
	41–45	13	6.4		Art and Publishing	15	7.4
	≥46	30	14.9		Others	36	17.9
Educational Background	High School	22	10.9	Year of Establishment	≤1	15	7.4
	Bachelor	84	41.6		1–3	44	21.8
	Master	53	26.2		3–5	29	14.4
	Doctor	15	7.4		5–10	30	14.9
	Others	28	13.9		≥10	84	41.6

### Measures

#### Extraversion

For extraversion, we used the five-item scale developed by Costa and McCrae (1992) and Mooradian et al. (2011), which ranges from 1, “completely disagree,” to 5, “completely agree.” Cronbach’s alpha values for the (5-item) subscales were 0.886.

#### Neuroticism

Neuroticism is measured by a five-item scale developed in the previous research (Costa and McCrae, 1992; Mooradian et al., 2011). These items are measured on a five-point Likert-type scale, ranging from 1, “completely disagree,” to 5, “completely agree.” Cronbach’s alpha values for the (5-item) subscales were 0.894.

#### Individual Creativity

Entrepreneurs’ individual creativity is measured as a set of personality traits including broad interests, autonomy, and preference for idea generation and divergent thinking (Amabile et al., 1996). To measure individual creativity, respondents reported on a five-item scale, ranging from 1, strongly disagree, to 5, strongly agree.

#### Individual Entrepreneurial Orientation

Individual entrepreneurial orientation is measured by a ten-item scale developed in the Bolton and Lane’s research (2012). This ten-item measure has three subscales of four items for innovativeness (Cronbach’s a = 0.761), three items for proactiveness (Cronbach’s a = 0.759) and three items for risk-taking (Cronbach’s a = 0.755).

#### Control Variables

Control variables refer to those variables that affect the results other than independent variables. In the empirical analysis, we use gender, age, industry, education level and years of establishment as control variables.

### Common Method Variance and Non-response Bias

In this study, we use Harman’s single factor test to rule out the common method variance, and use the principal component analysis to analyze the items of all variables to obtain the factor variance interpretation rate without rotation (Podsakoff et al., 2003). The results showed that the first principal component factor accounted for 37.507% of the total variance, which was lower than 40% of the critical value, and it did not explain the majority of variance, indicating that there was no serious common method bias problem in the survey data of our study, therefore had little impact on the subsequent analysis.

For non-response bias, we conducted the independent sample *t*-test on 62 invalid questionnaires and 202 valid questionnaires. The results showed that all *t*-values were not significant (*P* > 0.05), indicating that there is no need to worry about non-response bias.

### Reliability and Validity

#### Reliability

In this study, the Cronbach’s alpha and factor loading of a construct were used to test the reliability of the scale. As shown in Table 2, the Cronbach’s alpha of the variables involved in the study is higher than the standard of 0.700, and the combined reliability (CR) is higher than the critical value of 0.700, indicating that the scale in this study has a high level of reliability.

**TABLE 2:** Validity and reliability of construct measures (*N* = 202).

Item	Item wording	Factor loading
Extraversion(*a* = 0.886, CR = 0.8926, AVE = 0.6278)		
EX1	I’m talkative.	0.607
EX2	I’m confident.	0.794
EX3	I’m energetic.	0.798
EX4	I’m gregarious.	0.862
EX5	I’m sociable.	0.872
Neuroticism(*a* = 0.894, CR = 0.9012, AVE = 0.6481)		
NE1	I can’t bear too much pressure.	0.673
NE2	I always feel anxious.	0.762
NE3	I have a mood fluctuation when dealing with things.	0.885
NE4	I’m not stable, and my mood is always good and bad.	0.828
NE5	I’m easily irritated.	0.859
Individual creativity(*a* = 0.960, CR = 0.9601, AVE = 0.8281)		
IC1	I often look for new creative elements and inspiration and apply them to my work.	0.909
IC2	I am not afraid to take risks.	0.905
IC3	I usually suggest new ways to achieve goals and objectives.	0.913
IC4	I often have a fresh approach to problems.	0.912
IC5	In general, I am a good source of creative ideas.	0.911
Innovativeness(*a* = 0.761, CR = 0.8112, AVE = 0.5304)		
IN1	I often like to try new and unusual activities.	0.651
IN2	I prefer a unique approach to my work, rather than the reliable methods I’ve used before.	0.983
IN3	When I learn new things, I prefer to try my own unique way.	0.643
IN4	I prefer experimental and original solutions to problems rather than methods that others have used.	0.564
Proactiveness(*a* = 0.759, CR = 0.7892, AVE = 0.5605)		
PR1	I tend to plan ahead.	0.678
PR2	I prefer to take the initiative to wait for others to finish.	0.650
PR3	I prefer to act in anticipation of future problems.	0.894
Risk-taking(*a* = 0.755, CR = 0.8121, AVE = 0.5991)		
RT1	I am able to venture boldly in the unknown.	0.967
RT2	I’m willing to spend a lot of time or money on things that may yield high returns.	0.645
RT3	I tend to be bold when it comes to risk.	0.668

*c^2^/df = 2.190, RMR = 0.039, GFI = 0.843, AGFI = 0.804, IFI = 0.917, TLI = 0.903, CFI = 0.916, RMSEA = 0.077.*

#### Validity

Amos 21.0 was used for confirmatory factor analysis to directly test the validity through confirmatory factor analysis for the maturity scale. The results showed that χ^2^/df (<3), CFI (>0.900), RMSEA (<0.08) and other indexes were all in a good range, GFI and AGFI were also in acceptable range (>0.800), and the fitting degree of the model was good (as shown in Table 2). The standardized regression coefficient of each item to its corresponding latent variable exceeds the critical level of 0.500, and the average variance extraction (AVE) value of all variables exceeds the critical value of 0.500, which has good convergence validity. In addition, when the square root of the average value of the variable is greater than the absolute value of its correlation coefficient with other variables, the scale has good discrimination validity. In the descriptive statistical analysis and correlation coefficient matrix shown in Table 3, the value on the diagonal is the square root of AVE, which is greater than the absolute value of the correlation coefficient in the row and column where it is located, indicating that the scale has good discrimination validity.

**TABLE 3:** Descriptive statistics and correlations.

Variable	1	2	3	4	5	6	7	8	9	10	11
(1) Gender											
(2) Age	−0.144*										
(3) Industry	0.203**	0.289**									
(4) Educational Background	0.069	−0.104	0.104								
(5) Year of Establishment	−0.065	0.372**	0.239**	−0.057							
(6) Extraversion	−0.034	−0.077	−0.042	−0.099	0.038	**0.792**					
(7) Neuroticism	0.126	−0.078	0.028	−0.073	−0.013	0.050	**0.805**				
(8) Individual creativity	−0.003	−0.112	−0.023	−0.095	−0.015	0.496**	0.536**	**0.910**			
(9) Innovativeness	0.054	−0.128	−0.003	0.012	−0.139*	0.271**	0.367**	0.571**	**0.728**		
(10) Proactiveness	0.018	−0.026	−0.130	0.026	0.043	0.310**	0.235**	0.550**	0.353**	**0.749**	
(11) Risk−taking	0.051	−0.073	0.094	−0.123	−0.015	0.243**	0.251**	0.585**	0.270**	0.297**	**0.774**
Mean	1.460	2.356	5.658	2.718	3.614	3.826	3.849	3.409	3.137	3.198	3.153
*SD*	0.500	1.394	1.997	1.186	1.400	0.529	0.543	0.720	0.874	0.891	0.930

*N* = 202, *p < 0.05 (two-tailed test), **p < 0.01 (two-tailed test). The data below the diagonal is the correlation coefficient, and the data in bold on the diagonal is the square root of AVE.

## Results

### Descriptive Statistics and Correlations

Table 3. presents the descriptive statistics and correlation coefficients for the variables in this study. The results show that individual creativity is positively related to innovativeness (*r* = 0.571, *p* < 0.01), individual creativity had a positive correlation with proactiveness (*r* = 0.550, *p* < 0.01), and individual creativity also displayed a strong positive correlation with risk-taking (*r* = 0.585, *p* < 0.01). These results preliminarily supported hypotheses 1a, 1b, and 1c.

### Regression Analysis

In this study, we used SPSS 22.0 to test the hypothesis proposed above by means of hierarchical regression. The results are shown in Table 4. Referring to the research of Yu et al. (2015), to reduce the deviation caused by multicollinearity, the independent variables and regulatory variables are centralized in this study, and the corresponding quadratic terms are obtained by using centralized independent variables. By incorporating these quadratic terms into the hierarchical regression analysis, the significance level of the regression coefficient of the dependent variable can be obtained to assess whether an inverted U-shaped relationship exists. Model 1 is a regression model between the control variables and individual creativity. Model 2 is a regression model between the control variables, independent variables and individual creativity. Model 3 is a regression model between the control variables and innovativeness. Model 4 is a regression model between the control variables, individual creativity and innovativeness. Model 5 is a regression model between the control variables and proactiveness. Model 6 is a regression model between the control variables, individual creativity and proactiveness. Model 7 is a regression model between the control variables and risk-taking. Model 8 is a regression model between the control variables, individual creativity and risk-taking. The results are shown in Table 4.

**TABLE 4:** Results of regression analysis.

Variables	Individual creativity		Innovativeness		Proactiveness		Risk-taking	
				
	Model 1	Model 2	Model 3	Model 4	Model 5	Model 6	Model 7	Model 8
Gender	−0.021	−0.050	0.023	0.035	0.054	0.066	0.013	0.025
Age	−0.144	−0.045	−0.098	−0.016	0.001	0.083	−0.125	−0.042
Industry	0.029	0.042	0.049	0.033	−0.167	−0.183**	0.146	0.129
Educational Background	−0.110	0.015	−0.011	0.052	0.045	0.108	−0.153	−0.089
Year of Establishment	0.024	0.009	−0.114	−0.128*	0.088	0.075	−0.012	−0.025
EX		0.544***						
EX^2^		−0.416***						
NE		0.521***						
NE^2^		−0.350***						
IC				0.573***		0.566***		0.575***
R^2^	0.026	0.851	0.029	0.349	0.028	0.340	0.042	0.364
Adjusted R^2^	0.001	0.844	0.005	0.329	0.003	0.320	0.018	0.345
F change	1.033	122.030***	1.188	17.423***	1.110	16.740***	1.734	18.611***

*N* = 202, *p < 0.05 (two-tailed test), **p < 0.01 (two-tailed test), ***p < 0.001 (two-tailed test).

**FIGURE 2:**
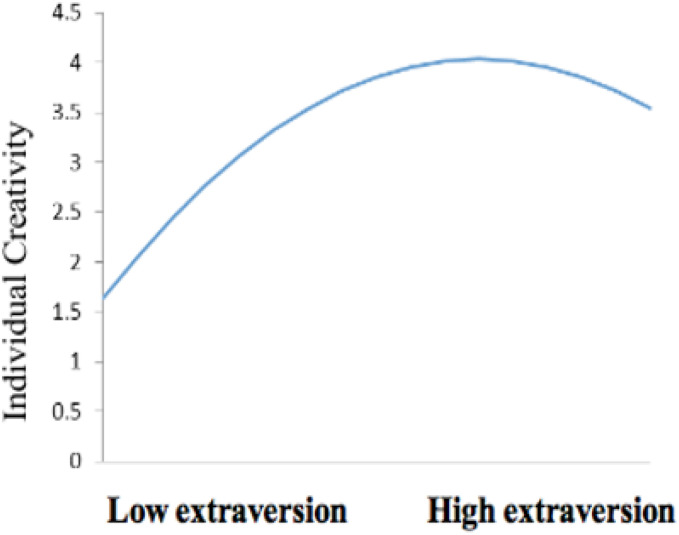
The Inverted-U relationship between extraversion and individual creativity.

**FIGURE 3:**
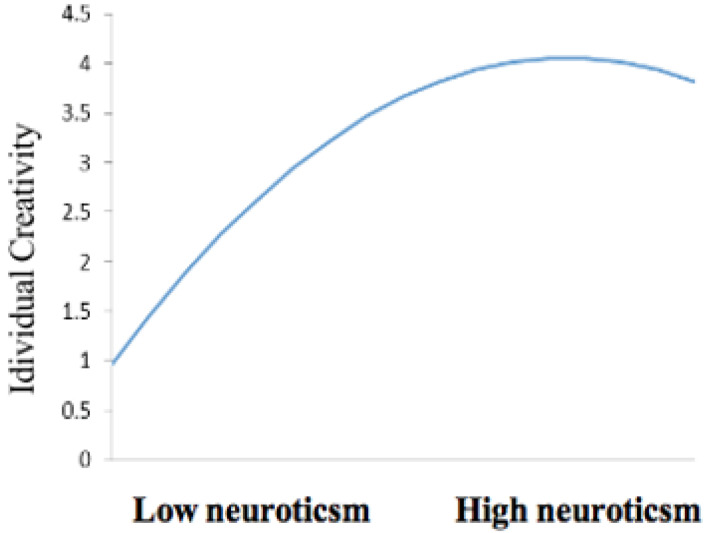
The Inverted-U relationship between neuroticism and individual creativity.

Model 2 showed that the square of extraversion and neuroticism had significant negative effects on individual creativity (β = −0.416, *p* < 0.001; β = −0.350, *p* < 0.001), indicating that neuroticism and extraversion exhibited a negative U-shaped relationship with creativity. These results confirm the validity of H2 and H3. Referring to the research of Cohen et al. (2003), the extreme point of the inverted U-shaped curve (i.e., curve vertex) appears at *x* = −b/2a, where x is the independent variable, a is the non-standard coefficient of its square term, and b is the non-standard coefficient of its primary term. Accordingly, the inverted U-shaped relationships between extraversion and individual creativity and the inverted U-shaped relationships between neuroticism and individual creativity are obtained (as shown in Figures 2, 3). According to the calculation, the extreme point of the inverted U-shaped curve between extraversion and individual creativity is located at 4.248 for extraversion and 4.032 for individual creativity. The extreme value of the inverted U-shaped curve between neuroticism and individual creativity was 4.382 for neuroticism and 4.060 for individual creativity.

In addition, the results of model 4 showed a significant positive correlation between individual creativity and innovativeness (β = 0.573, *p* < 0.001), which supports the validity of H1a. Model 6 showed that there was a significant positive correlation between individual creativity and proactiveness (β = 0.566, *p* < 0.001), thereby showing the validity of H1b. The results of model 8 showed that there was a significant positive correlation between individual creativity and risk-taking (β = 0.575, *p* < 0.001), confirming the validity of H1c.

## Discussion

Creative industries are characterized by the labor input of creative individuals and promote regional and national innovation and economic development, but it is still a relatively under-researched industry (Bilton, 2007; Yuan et al., 2019). Because creative industries are mainly composed of small startups, according to the upper echelons theory, the strategic choices and entrepreneurial behavior of creative entrepreneurs are crucial to the survival and development of enterprises (Chaston and Sadler-Smith, 2012). Therefore, this paper discusses the key driving factors behind the entrepreneurial behavior of entrepreneurs, including the influence of personality characteristics and creativity on their entrepreneurial orientation, and proposes an integrated model of the process from creativity to entrepreneurship at the individual level of creative entrepreneurs to predict their entrepreneurial behavior.

First, our research emphasizes the importance and particularity of creative entrepreneurs’ individual entrepreneurial orientation in creative industries and proposes and tests the influence mechanism of the structure of individual entrepreneurial orientation and its antecedents through empirical methods, thereby enriching relevant research on individual entrepreneurial orientation. Creative entrepreneurs’ behavior is mainly driven by their entrepreneurial orientation. Although the importance of entrepreneurial orientation at the firm level to entrepreneurial success is widely known, the literature lacks discussion at the individual level. Hence, this study considers that entrepreneurial initiatives might be implemented at different levels of aggregation (Goktan and Gupta, 2015; Wales et al., 2015), especially at the individual level (Bolton and Lane, 2012; Ferreira et al., 2015). Our research shows that creative entrepreneurs’ IEO in creative industries is different from that in other industries because the resource investment made by entrepreneurs in an extremely uncertain environment is to create symbolic recognition, not material interests. Therefore, their choice of entrepreneurship is to be able to embody their own interests, skills and talents in their work and to devote themselves to art. In addition, the existing research focuses more on the impact of entrepreneurial orientation on entrepreneurial success and firm performance, whereas there is still a certain gap in the research on the mechanism of how individual entrepreneurial orientation is produced (De Clercq et al., 2010; Ferreira et al., 2015; Gupta et al., 2016). Therefore, we continue to explore the impact of creativity on IEO. The results show that creative entrepreneurs’ creativity has a significant positive impact on the innovativeness, proactiveness and risk-taking of their IEO. To explore and develop new ideas, entrepreneurs must lead enterprises to adopt active and innovative strategies. The stronger the creativity of entrepreneurs, the more willing they are to break the paradigm, and the more likely they are to take risks in entrepreneurship. Second, we propose the individuality of neuroticism and extraversion exhibited a negative U-shaped relationship with creativity, showing that moderate extraversion and neuroticism are the best ways to stimulate creativity. Excessively high/low extraversion or neuroticism will have a negative impact on creativity at the individual level. This conclusion expands our understanding of the role of personality in creativity and explains the controversy regarding the influence of personality characteristics on creativity. Existing studies have reached different conclusions on the effects of neuroticism and extraversion on creativity in the big five personality traits, among which some researchers reported positive effects (Batey et al., 2010), others reported negative effects (Guo et al., 2017; Zhang et al., 2017) and some researchers reported no impact at all (King et al., 1996). In our research, we propose and test the non-linear relationships between neuroticism and creativity and between extraversion and creativity by referring to the TMGT effect and the “ubiquitous U” principle in the field of psychology and explain the variability of previous research results. This also provides new empirical support for the TMGT effect. Third, our research enriches the interdisciplinary research of entrepreneurship theory and psychology. To explain the reasons for the differences in entrepreneurial behavior and the results of entrepreneurs in creative industries and to reveal the psychological process behind entrepreneurial behavior, we proposed an integrated model of the process from creativity to entrepreneurship at the individual level by referring to the theories of big five personality and creativity. This model explains the influence mechanism of the key driving factors for successful entrepreneurship and entrepreneurial career choices. First, for the process from creation to creativity: moderate extraversion and neuroticism are the best ways to stimulate the creativity of entrepreneurs in creative industries, whereas excessively high or low values will reduce creativity. Second, for the process from creativity to entrepreneurship: the stronger the creativity of entrepreneurs in creative industries is, the greater the driving force for entrepreneurship. The entrepreneurial motivation and strategic tendency of creative industry entrepreneurs are different from those of entrepreneurs in other industries. Creative entrepreneurs choose to start a business to create symbolic recognition, and they are more inclined to realize their own creativity in an uncertain environment.

From a practical point of view, our study suggests that the important role of individual entrepreneurship orientation should be paid enough attention to by new ventures, which not only helps entrepreneurs to have a better evaluation of themselves to support their entrepreneurial process and results, but also helps to provide investment information for the business incubators and potential investment institutions. In addition, our results show that there is an inverted U-shaped relationship between extraversion, neuroticism and creativity. Therefore, entrepreneurs should attach importance to the evaluation and comprehensive understanding of their characteristics to improve their creativity, also, it is necessary to provide appropriate conditions for the creativity development of different types in order to shape and cultivate future entrepreneurs.

## Conclusion

For creative industries, the entrepreneurial behavior and strategic choices of creative entrepreneurs are very important for the survival and development of enterprises. Therefore, by combining the theories of personality, creativity and entrepreneurship, this paper proposes an integrated model of the process from creativity to entrepreneurship at the individual level of creative entrepreneurs to reveal the psychological process behind entrepreneurial behavior and predict entrepreneurial behavior and results.

Although the present study provides significant insights into this research topic, it also has several limitations. First, the industry distribution of samples is not sufficiently even; thus, the randomness of this sample must be reconsidered in the next study. Second, all the data in the empirical part of this study are static data from cross-section, and the survey design prevents a demonstration of causality and limits our ability to explore dynamic phenomenon, so longitudinal survey needs to be conducted in the future study to obtain time series data. Since contextual variables can moderate the curvilinear relationship between personality and creativity, future research needs to explore other potential moderating variables, such as cultural and social norms. Finally, it is not objective to use only self-assessment of leaders’ creativity. Future research using multiple measures of creativity (e.g., objective measures and psychological assessment) is needed.

## Data Availability Statement

All datasets presented in this study are included in the article/Supplementary Material.

## Ethics Statement

Ethical review and approval was not required for the study on human participants in accordance with the local legislation and institutional requirements. Written informed consent from the participants was not required to participate in this study in accordance with the national legislation and the institutional requirements.

## Author Contributions

YG and DZ: writing. YG and XD: providing idea. HM: providing revised advice. All authors contributed to the article and approved the submitted version.

## Conflict of Interest

The authors declare that the research was conducted in the absence of any commercial or financial relationships that could be construed as a potential conflict of interest.
